# Exploring the Needs and Perceptions of Iranian Families Faced with Brain Death News and Request to Donate Organ: A Qualitative Study

**Published:** 2012-05-01

**Authors:** Z. S. Manzari, E. Mohammadi, A. Heydari, H. R. Aghamohammadian Shearbaff, M. J. Modabber Azizi, E. Khaleghi

**Affiliations:** 1 *Faculty of Nursing and Medical Sciences, Tarbiat Modares University, Tehran, Iran*; 2 *Faculty of Nursing and Medical Sciences, Mashhad University, Mashhad, Iran*; 3 *Department of Psychology, Ferdowsi University, Mashhad, Iran*; 4 *Transplant Center, Mashhad University of Medical Sciences, Mashhad, Iran*

**Keywords:** Brain death, Organ donation, Emotional distress, Iran

## Abstract

**Background:** Learning that one of your beloved ones is passing away and you have to decide on organ donation is a very stressful experience.

**Objective:** To explore the specific needs of families with a brain-dead patient during organ donation process.

**Methods:** A qualitative research using content analysis was used to obtain data from 26 purposely selected families in a transplantation center in Mashhad, northeastern Iran, regarding how they would face organ donation decisions.

**Results:** Data saturation was reached after 38 unstructured in-depth interviews and field notes, once data was transcribed and tabulated. Four major themes emerged as 1) family needs for emotional support, 2) empathy and compassion, 3) team efforts to assure family, and 4) shouldering grief.

**Conclusion:** Study results highlighted the essential need for an expert team with specialized training to help families in despair deciding in favor or against organ donation. Moreover, discovering and explaining these specific needs help policy makers and administrators to plan interventions in relation to condition-building to facilitate safe passing of the families through this difficult situation.

## INTRODUCTION

Families faced with agonizing news of a brain-dead beloved one, experience high levels of stress and emotional turmoil with the decision to donate organs [1]. The recognition of family needs in such a complex situation by health care professionals is of paramount importance. Health care systems equipped to provide specialized services to these families, should be prepared to facilitate the decision making process regarding organ donation.

Although the issue of organ transplantation is significant worldwide, there has been few research studies conducted in the field, particularly in terms of nursing care duties as part of a team to assist the patient’s family [2]. Several published reports on this issue suggest the need for further studies in different populations and among families and individuals with diverse cultures [3].

For this study, researchers reviewed published works specific to the family needs when deciding to donate organ or decline, with an effort to identify the communication gaps between families and the health care team. Most studies reviewed were qualitative research in developed countries. In a study from England, researchers identified family needs for meaningful information from the nursing team and emphasized the importance of a better relationship between nurses and family members in such a grave situation [4]. The study of Chinese’ family needs living in Hong Kong, also reported the crucial importance of clear communication between the health care providers and families of a brain-dead patient to meet their emotional needs [5]. These two studies conducted in two countries with vast cultural differences, indicate that organ donating families have specific needs which though might not be identical in essence, are very important. The family emotional needs must be realized in different contexts and from global perspectives [6].

Eckenrod has determined that nursing team should have an expanded and re-defined role [7]. Data derived from thorough studies have highlighted the family needs for emotional support when deciding to donate organ or decline. Provisions must be made to ensure access to specialized care for families. It is necessary to explore the experience and knowledge related to the family needs for their patients [8].

Annually, there are 15,000 accidental brain-dead cases in Iran, of which, less than 10% of the families agree to donate organ at a ratio of 1,000,000:2, while many people die in need of organ transplant. Among the reasons provided for such a low rate of organ donation in Iran compared to the world statistics is the fact that organ donation is relatively a new concept and its multidimensional aspects have not been explored and clarified. This is an important issue with socio-cultural implication. Therefore, a systematic review and qualitative approach deemed necessary to investigate family perspectives and their emotional needs when caring for a brain-dead patient and experiencing a major decision in favor of or against organ donation.

## MATERIALS AND METHODS

Design 

Given the purpose of the present research which was to discover the family needs of a brain-dead patient in the decision making process for organ donation, a qualitative approach was selected to explore an understanding of human emotional needs, feelings and daily experiences [9], using content analysis approach.

Sampling and Setting 

Participants were 53 family members of brain-dead patients divided into two groups: One whose patient’s death occurred three months earlier and had consented to the organ donation, and another group consisted of families whose patients’ death occurred six months earlier and declined to donate organ. Of the 53 participants who agreed to be interviewed, there were 11 couples (parents), one sister, and three brothers as well as the spouses and children of the deceased patients who were married. The location choice for interview was made by the participants who mostly preferred their homes. A purposeful sampling method was used which included participants who had already witnessed brain death and organ donations and were willing to retell their experiences. This study started in July 2008 and ended in December 2010. The sampling continued until data saturation was reached or until no new codes were derived in the three final interviews. Therefore, 24 unstructured face-to-face in-depth interviews with open-ended questions and 14 semi-formal interviews (totaling 38) were conducted. Interview invariably lasted about 1–3 hours.

Data Analysis 

Content analysis of transcribed data was done immediately after each interview; field notes with nonverbal gestures such as crying, smiling, sighing and being silent were also incorporated. Written interview texts were read and re-read. Data were translated into units per meanings and formatted per context. The units of meanings were reviewed several times and encoded to be categorized according to the content similarity. Encoded data were minimized and compacted throughout the unit analysis process until the main and sub-categories were identified. At last, main categories with general and meaningful contexts determined themes. Necessary content modifications for each category were made and the analysis process was repeated. Irrelevant expressions were omitted from the interviews [10] (Table 1). Researcher kept field notes from the beginning to have records and insights to the process and later facilitate data analysis.

**Table 1:** An example of analysis process

Story	Code	relevant category	relevant theme derived from categories
I said to my doctor, “I’m for the organ donation itself, but what I want you to do before I make up my mind is to convince me into believing that there’s no other way… When I am not sure and have doubts even a bit I can’t make my decision… I really didn’t get you…” I didn’t tell him, but I wish I had asked him for his contradictions. (the participant did not consent)	-The effect of the family’s certainty of non-recovery of the patient on the facilitating decision making -Uncertainty in decision making due to doubt about recovery -Insufficient explanation by the physician in convincing the family -The importance of the certainty of the organ-asking physician’s about the family’s understanding of the situation and the elimination of the family’s ambiguity and questions	Necessity for counseling and giving information	Ensuring

Ethical Considerations 

The Ethics Committee of Medical Research, Tarbiaat Modaress University, approved the proposal and the informed written consent for this study. Volunteer participants agreeing to be a part of this study signed an informed written consent. Participants were assured of their safety, anonymity and confidentiality. Refusal to take part in the study or withdrawal at any point posed no harm to participants or their families.

Data Credibility 

To maintain data efficacy and credibility, manuscript was reviewed and modified with each unit analysis along with the extracted implications using supplementary participant views and suggestions. In addition, two qualitative researchers supervised and evaluated the entire study process. Furthermore, conformability, credibility, and transferability of data were established using triangulation method with maximum variance sampling in which, there were interviews with people of varying age, sex, social status, education, and relation to the deceased. Other factors which increased the credibility of the research, included spending sufficient time to conduct the study through open discussion and developing close relation with participants who felt at ease to express their hidden worries and find emotional some relief [11].

## RESULTS

Data analysis from interviews and the field notes produced 497 codes, 16 categories, and 4 themes. The identified themes included 1) family needs for emotional support, 2) empathy and compassion, 3) team support and family assurance, and 4) sharing sorrow.

Theme 1: Family needs for emotional support

By emotional support, family members meant someone paying attention to their psychological needs and offering sympathy to the family. From this theme five categories emerged: 1) receiving contradictory and unconventional news on the brain death status, 2) insufficient empathy when requesting donation of organ, 3) painful moments of harvesting organs, 4) tension with decision announcement, and 5) doubts after the patient’s death.

The emotional and psychological needs of the family are one of the main points repeatedly expressed. Families needed more support and empathy at the time of being informed about the brain death status and immediately asked to donate organs. Participants expressed their expectations for more understanding both on the side of the medical team who gave the news of brain death condition and on the part of physicians who were requesting for donation. Although, at the time families were informed their patient was brain dead, families requested for professional help from a psychologist, clergyman or peer support group, their needs were left unmet. The inhumane and cold manner of announcing brain death news and immediately requesting families to donate organs was shocking for most families for the level of physician’s indifference and apathy. A non-consenting participant (#5) who was informed of his child’s brain death status without prior attempts to assess his emotional state said: 

I am upset for the way I was told the news of my child’s brain dead so quickly and indifferent (crying profusely) when a little bird dies, one tries to explain and give the news with a long introduction by saying “maybe it escaped; maybe it is…” and other possibilities for the bird owner not to suffer emotionally. How many hours earlier was it? May be just 12 hours after my child was hospitalized… But the doctor emotionlessly and indifferently said, “your child’s brain cells have died…”

Empathy expression by the medical team was found helpful to families who needed emotional support by people who understood their grave situation. Show of compassion helps reduce tension and assist families make the right decision. For most families, the most difficult time was the period between signing a consent form and the actual transfer of the body to the operating room for organ harvesting. All the families experienced agony and severe emotional distress during this period. They knew their decision was made to save someone’s life and yet, end another. Participants described these moments as bitter, hard, and unbearable and wished this period could have been reduced and the body of their beloved one returned to them in the shortest time post-surgery. Families greatly needed emotional and psychological support during this stressful period as a consenting participant (#2) stated below:

At that moment, parents are thinking that their child is being slaughtered into pieces. This is a hard decision to make. The hardest and most hurtful time for me was the 24-hour period that afternoon when I made my decision until the actual transplant took place. It was very hard on me (weeping loud). This 24 hours were hell for me (sobbing). At any moment I thought my child may return, but I had already consented (crying). If that time was shorter…

Family distress from the decision to donate or decline did not end after the patient’s death. The emotional torment continued due to a lengthy official process without any follow up or psychological support for the family members as a non-consenting participant (#4) added: 

Although I didn’t consent, the thought of such a request…, and thinking to myself how much being pressured and coarse by others bothered me? We shouldn’t leave families unattended. We should follow up and ask questions.

Theme 2: Empathy and compassion

What all the families mentioned as their most fundamental needs was an improved communication pattern and humane behavior between health care providers and family members during patient hospitalization, far before the brain death state is announced and the way a family is notified and pursued for organ donation.

The only thing that I wanted was proper and clear communication. A human relationship with the family is very important (the consenting participant #1).

This theme generated three sub-themes: 1) believing in the process to decide in favor of organ donation, 2) developing a trusting relationship with the physicians, and 3) understanding the emotional paradox of contrasting messages. All the families wanted to have a better relationship with nurses throughout the process. Families considered trusting relation with the health care team as an important and positive step to help them acceptance the brain death condition and accelerate the process in favor of organ donation. A consenting participant (#5) shared:

At that moment, we were needed a calm and trustworthy person with a sincere sense of sympathy and kept looking for that person among the doctors and nurses. Such a person could have made it easier to accept the news and decide on organ donation. If they can not behave that way, it drives us mad; it doesn’t mean we will stop thinking about the decision; in fact, we were annoyed and irritated. However, their manners have a strong effect on the family and convey that they are not fully engaged in their job…

A compassionate and trusting relationship with the physicians and nurses can improve the participatory spirit and assure the family to make efforts along with the medical team without worries that more could have been done to rescue the patient. Improper behavior by any member of the health care team can create family mistrust in the system with negative feelings toward the donation process as a non-consenting participant (#5) said: 

I needed their understanding and attention. I needed them to communicate with me during that hard situation. But their cold and inspirited way of treating me had a negative effect on my morale and belief system. I didn’t trust them…

Some families noticed behavior changes in personnel after they decided in favor of or against the organ donation. Families who signed a consent form in favor of donation were treated with respect, their request for visiting their patient was honored and their questions were positively answered. While families who declined to donate organ, noticed a negative attitude and an abusive behavior from the health care team who did not reach their organ donation goals. The non-consenting participant (#7) indicated:

It’s interesting that just before the issue of donation request was raised, nobody spoke a word to us; they didn’t let us even see our patient in person, but as soon as we favored the organ donation, everything changed suddenly. Everyone was suddenly kind, and good mannered; they let us go to see our patient. Wouldn’t you call this abusive and coercion? 

Theme 3: Team efforts to assure family

Family assurance and support by the medical team means giving families a sense of comfort by providing sufficient and proper information on the patient’s condition. The main focus of this theme was family support by the physician in charge and the nursing staff who asked for organ donation. Five sub-themes emerged: 1) certainty of the brain death diagnosis, 2) test accuracy to confirm the brain death status, 3) religion’s approval and acceptance of organ donation, 4) family counseling and provision by a trusted physician, and 5) reinforced accuracy of provided medical information.

Most families needed to know that a brain death state was irreversible based on the patient’s apparent features such as breathing and heart beat. Families wanted a clear understanding of their patient’s condition which could have been facilitate to accelerate their decision making process. They needed a transparent and comprehensible report on a definitive, irreversible and incurable brain death condition beyond any possibility for recovery or return to normal life. Families struggled when no one could confirm their patient was permanently brain dead and yet talked about organ donation. A non-consenting participant (#4) expressed: 

We had to make sure there was no return (emphasizing forcefully). They should have convinced me… The kid was still alive anyway, breathing even with the help of the apparatus. His heart was fine…

The information obtained by examining the patient to confirm a brain death condition was another part of the family dilemma as they repeatedly asked to see the test results (especially the brain scan and apnea test) before deciding to donate organ. Hospital regulations only tested and provided the results of a brain scan and apnea after the family has signed a consent form to donate organ. Families were annoyed and experienced much doubts and unhappiness regarding their decision to donate organ. The non-consenting participant (#1) stated:

Now, I am very sad and have to complain. Why did they put us under so much pressure? Why didn’t they tell us and show us the evidence? They didn’t show us even a simple brain scan result. If they’d talk to us with proof, or show us the result of the brain scan, we’d have decided differently… 

Families needed information about the organ transplant operation. They wanted to know if the surgery would be painless for a brain-dead person. Families wanted to know something about the identity of organ recipient without violating anyone’s privacy to make them feel a little at ease. Families described the importance of access to the physician in-charge to explain them some of the processes involved. They needed reassurance about the whole process. Participant (#1) who did not consent said: 

I wanted to talk to the doctor so many times. Those days, that was the top priority for us. I wanted him to make us feel some relief; I mean I wanted him to tell us his diagnosis was right and the patient wouldn’t return. This way I could think of organ donation.

Access to information and reassurance on the accuracy of diagnosis was repeatedly mentioned by families as their fundamental need. Families continued to wonder even after the donation process has ended. Once the organ donation process and burial have occurred, for many months families experienced doubts and uncertainty about their loved one being truly brain dead. Among the families who had consented to donate organ, this issue appeared in form of severe mental anguish. They experienced doubt because of contradictory information, had negative feelings for a hasty decision, felt guilt and sorrow for a sense of failing someone who could have survived and lived. A consenting participant (#3) said: 

I sometimes tell myself “maybe I was rushed; maybe he could have returned; maybe he would have recovered and we didn’t let him…”

Theme 4: Shouldering grief

Humans are needed to be understood when grieving, expressed by families who mourned. The three sub-themes identified were: 1) support during the official procedures, 2) the healing effects of health care team being present at funeral services, 3) honoring the good deed by the family and the deceased. Families wanted a quicker process for official and legal proceedings for the delivery of body to the family for burial. Hospital services which included cleaning and transferring the body to the family was followed by a large volume of legal procedures. Attempts were made to reduce family stress and hardship by accelerating some of the functions. These services, however, were only offered to families who consented to donate organ and still some of the donating families remained dissatisfied with the hospital services; consenting participant (#6) shared: 

It was hell for me at the forensic medicine office—I just stayed there. It was a bad time (with emphasis); I was kept waiting so long that I said “either give me the body, or I will leave.” I was ready to give up everything.

Hospitals should develop policies requiring health care team members to be present when a body is being delivered to the family in order to offer emotional support. Having a member of the medical team present at the funeral, to offer the family condolences would be not only a great emotional support but, a kind gesture to offer respect to the family for the decision they made. A consenting participant (#14) said: 

It was very good that some people from the transplant center came to the funeral ceremony. It was comforting to me… I felt they respected our decision…

Participants voiced their expectations and desires for honoring their deceased and recognizing the organ donors in some form. Among the suggestions were a memoriam, a plaque, a monument, a designated burial site, an engraved tombstone, and holding memorial ceremonies to commemorate them for their generosity in the presence of donor recipients. A participant (#2) who consented said:

Unfortunately, we received no attention. What’s wrong with having a monument for these people? If we are in a small city, a hospital can be named after the donor. They are not any less than a martyr.

## DISCUSSION

The result of this study highlights many important issues related to families of a brain-dead patient struggling with the decision of donating organ. Family experiences at an emotionally charged event identify their needs for support, empathy and compassion, team efforts and family assurance, and shouldering grief. The implications for practice emphasize the necessity to make appropriate changes at various levels. Findings of this study are comparable to those found in studies from other countries.

Literature reviews have identified basic human needs for emotional support, clear communication, and information offering to relieve doubt and anxiety. The core findings in this study which is different from other studies relate to shouldering grief. No other study has mentioned the distinctive human need discovered in this study mainly attributed to the Iranian culture with a system where social and religious ceremonies are highly valued. 

The concept of emotional support, according to Sanner refers to family needs for sympathy and support by nursing and medical staff in order to help them calm down during their preliminary reaction to the brain death news [12]. Receiving this support could help families face the situation logically. In Sanner’s study, families who were given information and assurance by the nursing staff and physician in-charge, agreed to donate organ. Similarly, this study found family support to be essential for relieving concerns and misunderstandings especially when the body is moved to the operating room for organ harvesting and family stress mixed with tension and conflicting emotions leads to the perception of ending a child’s life by slaughtering his body for another person to survive. Clear and timely information are essential to support a family during a life altering occasion to make the best decision. 

In a qualitative study by Sque, *et al* [13], researchers revealed that family’s inclination to support the patient may cause a contradiction between donating life to others and sacrificing their own patient. In another study by Sque in England [14], feeling guilty and at the same time love for humankind was a common experience among family members. Here, we concluded that before the issue of donation is introduced, the family of a brain-dead patient should be offered emotional support and psychological consults. It is necessary to prepare families at such difficult times and avoid further emotional injuries. One of the most expressed family needs was presentation of sufficient data to support the diagnosis of brain death. Families repeatedly asked for a clinical test and evaluation showing the patient was indeed brain dead before asking for organ donation. To conduct clinical tests for determining brain responses families must sign a consent which automatically showed an agreement to donate organs. This was a vague and challenging process for some families. Families in doubt said they would have consented to donate organs if they had known with certainty the test results confirming presence of no brain activities and assured of brain death condition.

Ormrod, *et al*, investigated family comprehension of brain-dead patients for their grief reactions and different attitudes towards organ donation especially among those who had seen the test results on brain activities [15]. Researchers found that many families understood when the test confirmed no brain activity; there was no more hope for their patient to live. All the families who consented to organ donation understood the meaning of brain death. Seven families said that seeing the brain test results helped ease their decision for organ donation. Considering the results of current study, it seems essential to assure families by providing clear information particularly regarding brain function. Although this is a common practice in most medical centers, in certain situations, families could be severely affected emotionally and cognitively and information alone may not be enough. Rather, with respect to the ambiguities and misunderstanding among families, it would be important to pay attention to the manner in which information is provided and reiterated. In line with the findings of this study, Long, *et al*, revealed that most families faced insufficient information to reach proper comprehension of brain death concept even after the patient has died [4]. Thus, information must be provided with specific provisions and adequate content for comprehension. Besides verbal explanation, written materials and visual expression can be used to boost comprehension. 

Results of our study indicate that family counseling must be offered before brain death status has been revealed. A meeting between the family, nurse and the physician would be essential to convey proper information on the patient status. Awareness on the disease process and prognosis gives families an opportunity to develop trust with the health care teams and better acceptance the brain death concept before discussing organ donation. 

Other family needs mentioned were seeing some one care for their patient and having the chance to visit their patient. Family assurance on receiving sufficient and proper care and attention can facilitate the decision to trust the team. Furthermore, families feel less tensions and mental struggles for proper care being offered to the patient which helps reduce sense of guilt after death. In another study, one of the family needs mentioned was adequate patient care which was assuring and reduces tension when patient’s basic needs were met [4].

Families needed to communicate their concerns regarding patient care with the health care team based on respect, and mutual affection for the patient. Communication was one of the most important emotional and mental needs for families in such a difficult and complicated situation. In fact, communication served as a bridge between the family and the team, and facilitated making decisions. Most families complained about the improper communication and contributed poor communication as a factor for disbelief and mistrust in the team. In a qualitative study, Kesserling, *et al*, showed that long memories of the mournful relatives were affected by the features of decision making (obvious versus vague) and the quality of the interaction that the team members had with them [16]. Another noteworthy result in this study was the distress and conflict experienced by family members who agreed or disagreed with the organ donation decision after the patient’s death. The family conflict was mainly focused on the negative experience of making decision without knowing the facts. Members of a family faced with incomplete and contradictory information had to make a life and death decision with all the uncertainties surrounding the brain death status as an irreversible condition and having the difficult task of differentiating one condition with another such as coma.

Studies have shown that a decision for or against organ donation without access to the test results can be stressful and provoke family conflicts. Therefore, families need support and follow up after their decisions and after the patients’ death. Haddow found that the decision to donate organs can cause anxiety and conflict [17,18]. Sque, *et al*, stated that dissonance, conflict and anxiety were the outcomes of decision making for organ donation [13].

In conclusion, similar to other studies in this field, our results show that more than anything, families require emotional and psychological attention. Although family needs from global perspectives show similar findings, the feature of shouldering grief during the mourning period seem unique and culture-based since mourning in the Iranian culture is a religious and social rite. Unfortunately, current medical and nursing services in Iran are inadequately prepared to meet the patient and family needs when confronted with organ donation decision of a brain-dead patient. Therefore, according to the results of this study, we suggest the formation of a hospital-based specialized team consisting of nurses and doctors to attend to family needs. The team should receive special training in communication, building trust, showing care and compassion and addressing family needs in such complicated and complex situations. They need training on providing emotional support with counseling skills.

It seems necessary to facilitate an official process with legal formalities to offer families the option of having brain function test results at hand before signing a consent form to donate organs. Moreover, provide services to prevent traumatic psychological impacts by accurate and clear information regarding their patient’s health status. Families should be treated with respect and dignity regardless of their decision to donate organ or not. Families should have access to their patients at all times and with or without donation the body should be returned to families for proper burial. Medical facilities should be monitored for unethical conducts when families are coarse to donate or else.
